# The effect of stress and exercise on the learning performance of horses

**DOI:** 10.1038/s41598-021-03582-4

**Published:** 2022-02-04

**Authors:** Cathrynne Henshall, Hayley Randle, Nidhish Francis, Rafael Freire

**Affiliations:** School of Agricultural, Environmental and Veterinary Sciences, Charles Sturt University, Wagga, Wagga, NSW Australia

**Keywords:** Neuroscience, Psychology

## Abstract

Domestic horses are widely used for physically demanding activities but the effect of exercise on their learning abilities has not been explored. Horses are also frequently exposed to stressors that may affect their learning. Stress and exercise result in the release of glucocorticoids, noradrenaline and other neurotransmitters that can influence learning. It is not currently possible to directly measure concentrations of neurotransmitters in the brains of behaving horses, however the inference of neurobiological processes from peripheral markers have been widely used in studies of human cognition. We assigned 41 horses to either ridden exercise, uncontrollable stress or inactivity and evaluated their acquisition of an industry-style aversive instrumental learning task. Exercised horses achieved the learning criterion in the fewest number of trials compared to the stressed and inactive horses whose performance did not differ. The exercised horses’ salivary cortisol concentrations decreased during learning whereas the concentrations of the other groups increased. Spearman’s correlations revealed that horses with the highest cortisol concentrations required the most trials to reach the criterion. We present novel data that exercise prior to learning may enhance the acquisition of learning in horses. Conversely, activities that expose horses to uncontrollable stressors causing strong cortisol release may impair learning. It is proposed that these effects may be due to the influence of neurotransmitters such as cortisol and noradrenaline on brain regions responsible for learning.

## Introduction

Domesticated horses are used for a wide range of physical activities requiring extensive training to allow control by the rider, driver or handler. The resultant behavioural responses are underpinned by complex neural processes that facilitate learning^1^. There are currently no methods for directly measuring concentration of neurotransmitters in the brains of behaving horses. Experimental work in the use of EEG in moving horses is in its infancy^2^ and the validated proxy for dopaminergic activity, spontaneous eye blink rate, requires horses to be stationary to enable accurate measurement^3^. Studies of human cognition provide suitable methodologies for inferring putative neurobiological processes underlying observed cognitive outputs based on peripheral concentrations of neurotransmitters (for example^4^). In the present study, we aimed to replicate this approach in horses, using peripheral concentrations of cortisol and a proxy for noradrenaline, heart rate (HR), to infer the putative neurobiological processes underpinning observed cognitive outputs in relation to exposure a stressor, exercise or inactivity.

Exercise is physical activity that leads to an increase in oxygen consumption, metabolic load and as a consequence, an increase in cardiac output as well as range of other physiological processes to support increased demands on the body^5^. The exercise physiology of horses has been well characterised^6^, and in other species, exercise has been shown to benefit learning, memory and cognitive functioning^7^. While the cognitive abilities of horses have been explored in a range of studies (reviewed in^8^), the potential effects of exercise on equine cognition have not been tested experimentally. In common with human athletes, domestic horses are routinely exposed to a physical warm-up prior to a training session or competition because of perceived physical and mental benefits^9^, however in one study, warm-up intensity was found to be negatively correlated with subsequent performance in a show jumping competition^10^.

Stress has been defined as an experience, context or stimuli perceived to threaten or disturb the homeostasis of an organism^11^. Stress encompasses a state of heightened arousal, a perception of aversiveness and a lack of control^12–14^. At the cognitive level, stress may induce a state of uncertainty about the most appropriate action to take to safeguard physical, emotional or mental well-being^15,16^. Stress exposure can be adaptive, causing beneficial modification to brain regions to support learning^17,18^ or maladaptive, especially when it can’t be escaped or controlled, leading to learning impairments and psychopathologies^14,15^.

There is growing evidence that exposure to different forms of stress can affect equine learning, depending on the stressor, type of learning task and in some studies, the temperament of the horse^19–21^. In a series of studies, exposure to repeated short-term uncontrollable stressors impaired working memory^19^, and appetitive instrumental learning and also had a tendency to impair aversive instrumental learning^20^, but a single 30 min stress exposure prior to learning transiently enhanced appetitive instrumental learning^21^. In comparison, a mild controllable stressor had no effect on aversive instrumental learning^22^. Collectively these findings suggests that equine cognition may be sensitive to the effects of uncontrollable stress. With the exception of Fenner et al.^22^, the learning tasks employed in these studies are not generally reflective of the types of cognitive tasks horses undertake in industry settings^23^, where apparent failures to learn can lead to risks to both human safety and horse welfare^24,25^. Consequently, there is a need for more research into the effects of stress and exercise on equine learning in industry type settings.

Of particular importance is the type of instrumental learning task used in studies of equine cognition, as this determines how closely the experimental setting mirrors industry conditions. During instrumental learning, behaviour that results in a beneficial consequence for the animal will be repeated. Beneficial consequences can include receiving something rewarding such as food or play, otherwise known as appetitive conditioning or positive reinforcement, or escaping from something perceived to be aversive, otherwise known as aversive conditioning or negative reinforcement (NR). The majority of studies in equine cognition with or without stress exposure use positive reinforcement learning protocols^23^, however NR is the instrumental conditioning paradigm predominantly used in horse training^26^. The effectiveness of NR relies on the use of aversive stimuli applied to areas of the horse’s body (such as the mouth or thorax) to cause it to cease a current behaviour^27^ and commence a new behaviour in order to escape the effects of the aversive stimulus. The termination of the stimulus after the new behaviour commences reinforces that behaviour, making it more likely that behaviour will be repeated in the future^28^. The aversive characteristics of the stimuli used in NR training may be perceived as a stressor, and this may affect learning, particularly during early trials before the horse has learned how to reliably or rapidly escape the stimulus by performing the desired response^29^. The predominant use of NR paradigms in industry settings makes it likely that a great deal of equine learning occurs under some level of stress. As horses are also frequently exposed to extrinsic stressors of varying intensity and controllability during a range of routine handling, housing, management and training activities^30,31^, it is possible that the cumulative effect of these exposures may negatively affect their acquisition or recall of NR learning. For example Christensen et al.,^32^ reported the retrieval of NR learning tasks was impaired in horses exposed to extrinsic stressors (novel objects and novel environment).

The physiological stress response encompasses two interconnected branches of the autonomic nervous system, the rapid responding sympathetic adrenal medullary (SAM) network which facilitates adrenergic release into the circulation and ultimately brain regions and the slower hypothalamic pituitary adrenal (HPA) network which facilitates glucocorticoid release^11,33^. Research in other species suggests the release of these neurotransmitters during stress^34–36^, along with dopamine, endocannabinoids, brain derived neurotrophic factor (BDNF) and serotonin^37–40^ mediate the effects of stress on learning. These neurotransmitters affect neuronal functioning in many ways, including changes to neuron excitability, morphology and complexity^41,42^ across many brain regions necessary for learning^43–46^. Stress effects on learning depend on the timing of the stressor (before, during or after the learning)^47^ and characteristics such as intensity^48^, duration^49^, controllability^50^ and predictability^51^. Controllable stressors, or those of low to medium intensity may cause moderate increases in concentrations of glucocorticoids and catecholamines prior to or during learning which are associated with enhanced performance in rodent and humans. In comparison, severe, uncontrollable stress exposure has led to the opposite effect^52,53^.

In common with moderate psychosocial stress, exercise has been shown to enhance cognition and memory in rodent and human subjects^54–56^. The brain mediated physiological response to exercise including patterns of neurotransmitter release shares some similarities with stress, such as the release of glucocorticoids and noradrenaline which are essential for the mobilisation and regulation of energy sources to enable responses to threats or physical activity as well as having benefits for cognition^57–59^. However, whereas stress exposure leading to increases in such neurotransmitters can impair learning, exercise even at levels of high intensity, is generally positive for learning in human and rodents^56,60,61^ and has been shown to counteract the negative effects of stress on learning^62–64^. The neurobiological mechanisms by which these divergent effects occur remains to be fully elucidated^59,60^. In equine exercise physiology studies, moderate to high intensity exercise results in an increase in adrenaline and noradrenaline concentrations in combination with increasing heart rates (HR)^65,66^ and exposure to a startle inducing novel object also resulted in increased adrenergic release at HRs considerably lower than those reported in exercise studies. Increasing peripheral concentrations of noradrenaline, such as occur in response to stress and exercise, in combination with corticotrophin releasing factor in the brain stimulate noradrenaline release in brain regions via the locus coeruleus^33,67^, ultimately influencing cognition^68^. In the equine stress literature, HR is frequently used as a proxy for sympathetic adrenal medullary system (SAM) activity^31,69–71^. Exercise also elicits the release of other neurotransmitters associated with cognitive enhancement, including brain derived neurotrophic factor (BDNF), endocannabinoids, dopamine and others^59,72,73^ and it is possible these substances are beneficial for equine learning during exercise, including under conditions of psychosocial stress, such as learning to carry a rider for the first time^74^.

**Figure 1:**
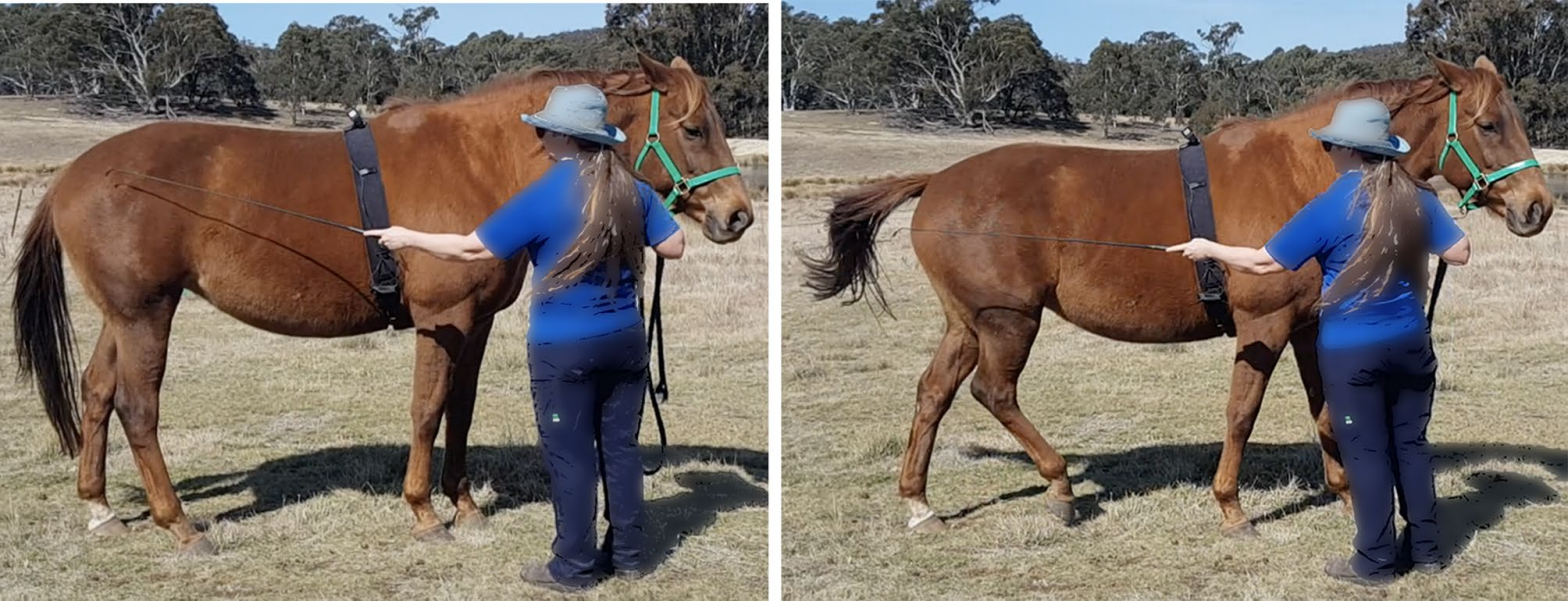
Learning task: Position of whip taps and reinforced response during learning task.

This study compared the effect of exercise, stress or inactivity prior to learning on the acquisition of a negatively reinforced learning task in which the horse was tapped with a training whip on its hindquarter until it made a sideways locomotory response (Fig. 1). After an inactive pre-test period (PT), forty-one horses (Supplementary Table 1) at three professional horse training facilities were assigned to either a calm ridden exercise session (E) unpredictable, uncontrollable stressor (S) or an inactive (I) treatment of 22 min duration that was sufficient to initiate responses from the HPA and SAM networks. We measured HR as a proxy for SAM activity (adrenergic release) and saliva and serum samples were collected to measure cortisol (HPA axis) and BDNF concentrations respectively. We hypothesised that the exercise treatment would enhance learning acquisition in comparison to the stress and inactive treatments. BDNF analysis is not reported due to the destruction of the serum samples prior to analysis as a result of the malfunction of a laboratory freezer. Data are reported as mean + 95% confidence intervals with statistical significance at *p* < 0.05.

## Results

### Number of trials to reach learning criterion

**Figure 2:**
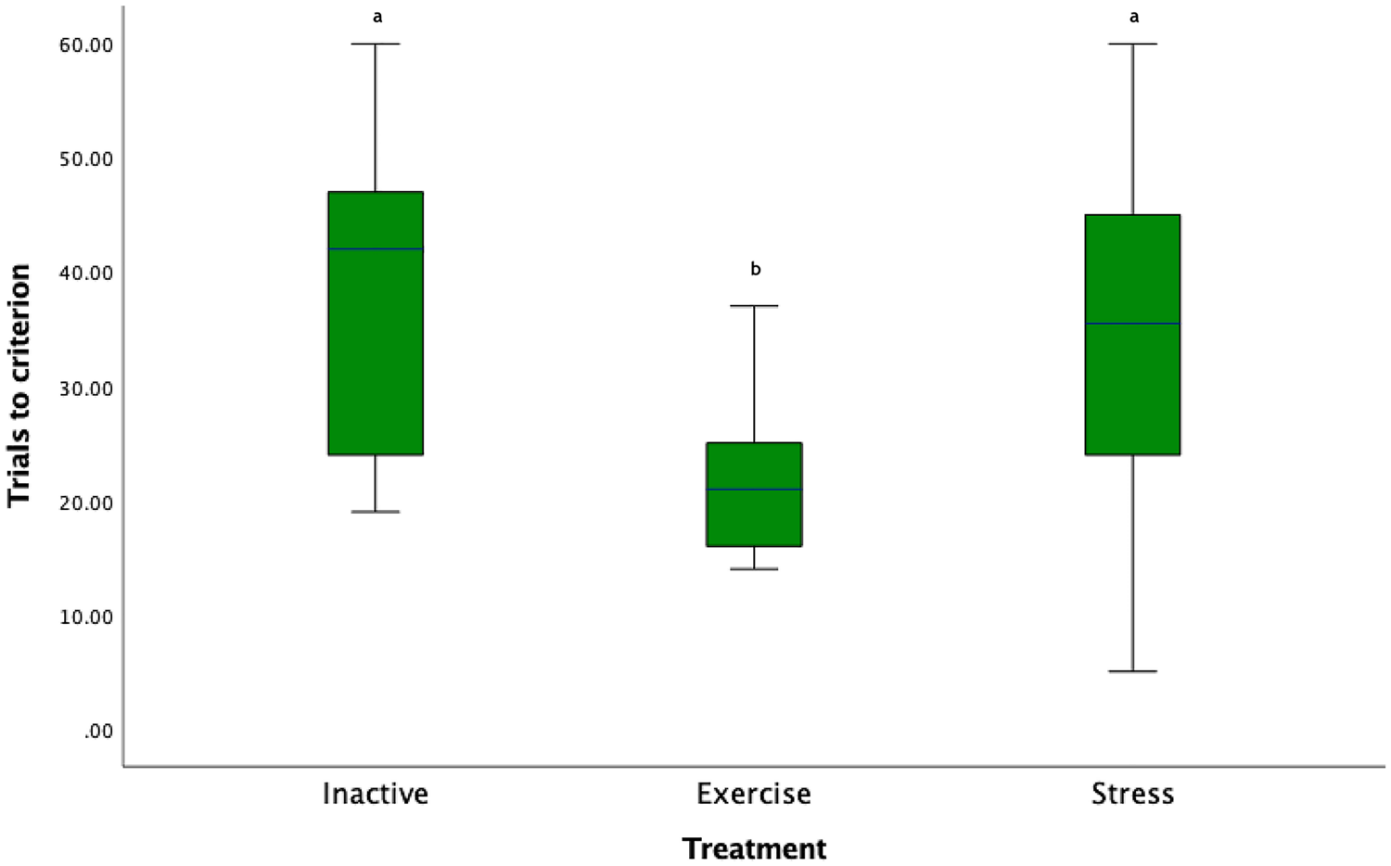
Box plot of number of trials to reach the learning criterion: Key: Letters that differ are significantly different at p < 0.05).

The number of trials to reach the learning criterion differed between treatment groups (χ^2^ (*41*) = 10.27 *p* = 0.006; Fig. 2) and pairwise comparisons with Bonferroni correction, revealed that the E horses required significantly fewer trials than I and S horses to reach criterion (E-I: (χ^2^ (*2*) = 14.04, *p* = 0.007, E-S: (χ^2^ (*2*) = −11.19, *p* = 0.046). There was no significant difference between the I and S horses (χ^2^ (*2*) = 2.86, *p* = 1.00). The number of trials to reach criterion did not differ between the three experimental locations (χ^2^ (*2*) = 0.44, *p* = 0.8).

### Other learning factors

There was no difference between the treatment groups in the rate at which they learned the correct response (χ^2^ (*2*) = 5.99, *p* = 0.05, post hoc results in Supplementary Materials). There was also no significant difference in the number of taps required to reach the learning criterion (χ^2^ (*2*) = 3.27, *p* = 0.195, I: 226.93 [140.81–313.05 95%CI], E: 135.31 [67.79–202.82 95%CI], S: 273.14. [73.79–472.50 95%CI]).

### Heart rate

**Figure 3:**
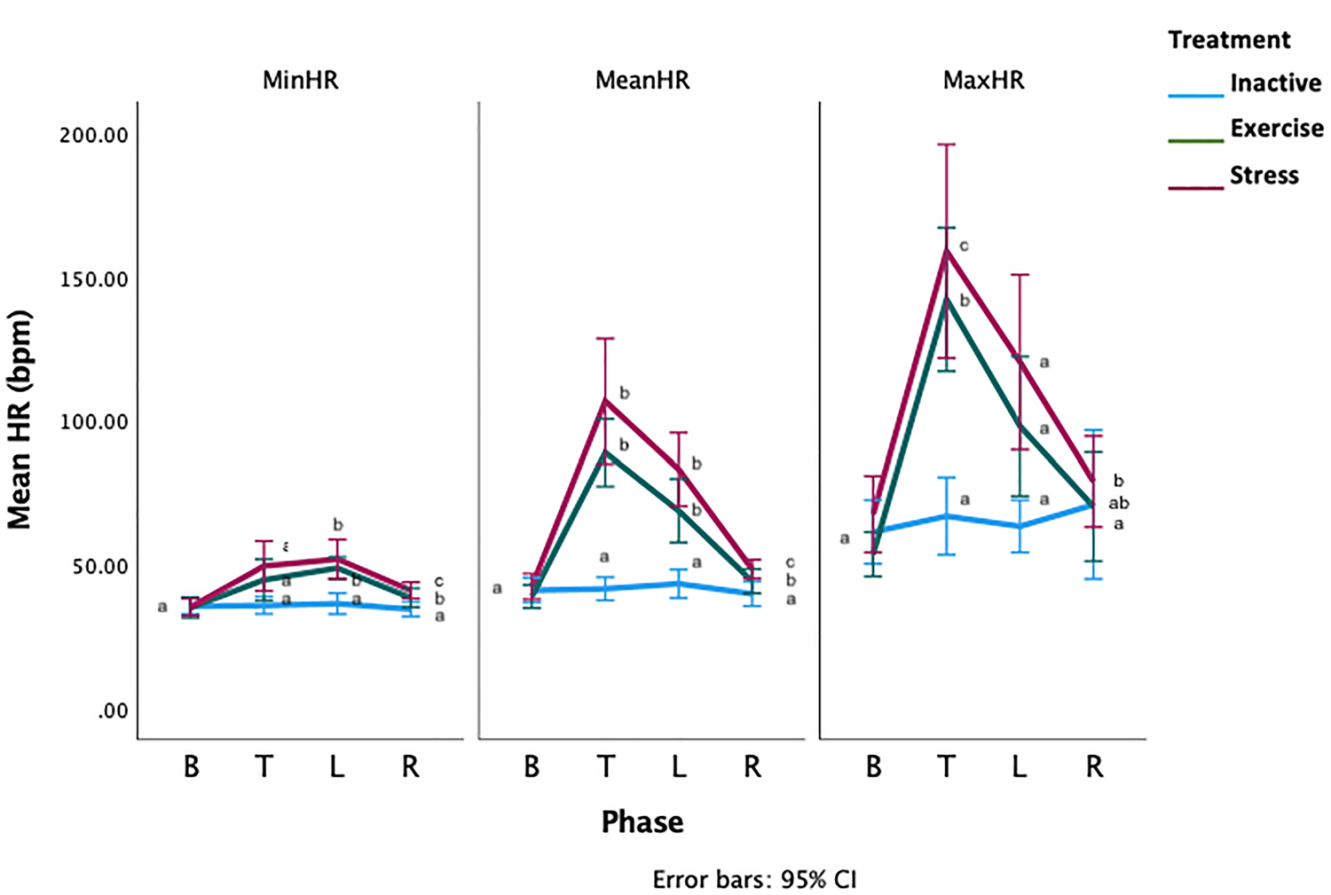
Means of the minimum (MinHR), mean (MeanHR) and maximum (MaxHR) heart rates for the PT = pre-test phase T = treatment phase, L = learning phase. Letters that differ within each phase differ significantly at p < 0.005. Phases without letters do not differ.

There was a significant phase*treatment interaction for the mean, minimum and maximum HRs (General linear model with repeated measures GLM-RM: Mean HR, F_2.16,41.16_ =9.88, *p*=0.000002, Minimum HR-*F*_2.9,55.8_ =4.88, *p*=0.005, Maximum HR- *F*_2.9,55.8_ =4.88, *p*=0.005, Fig. 3). During PT, the mean HRs were similar for all treatments (General linear model GLM: Mean HR: *F*_2,38_ =0.78, *p*=0.46). During T, the mean HRs of the S and E horses was significantly higher than the mean HRs of the I horses (GLM: Mean difference [bpm]: I-E: −47.31 [70.82–23.80 95%CI], *p*=0.00005, I-S: −65.21[88.28–42.13 95%CI] *p*<0.000001, E-S: −17.90 [41.40–5.61 95%CI], *p*=0.165). During L, the E and S horses had significantly higher mean HRs than the I horses (GLM: Mean difference [bpm]; I-E: −25.38 [41.57–9.19 95%CI], *p*=0.001, I-S: −39.76 [55.65 −23.87 95%CI] *p*<0.000001, E-S: −14.38 [30.57–1.81 95%CI], *p*=0.09, minimum and maximum HR data in Supplementary Materials). There were no significant phase*location interactions for any HR measure, (GLM-RM: Mean HR: *F*_3.1, 59.8,_ 0.18, *p*=0.914, Minimum HR: *F*_3.7, 70.7,_ =1.43, *p*= 0.236, Maximum HR: *F*_4,66_ =1.43, *p*=0.2).

**Figure 4:**
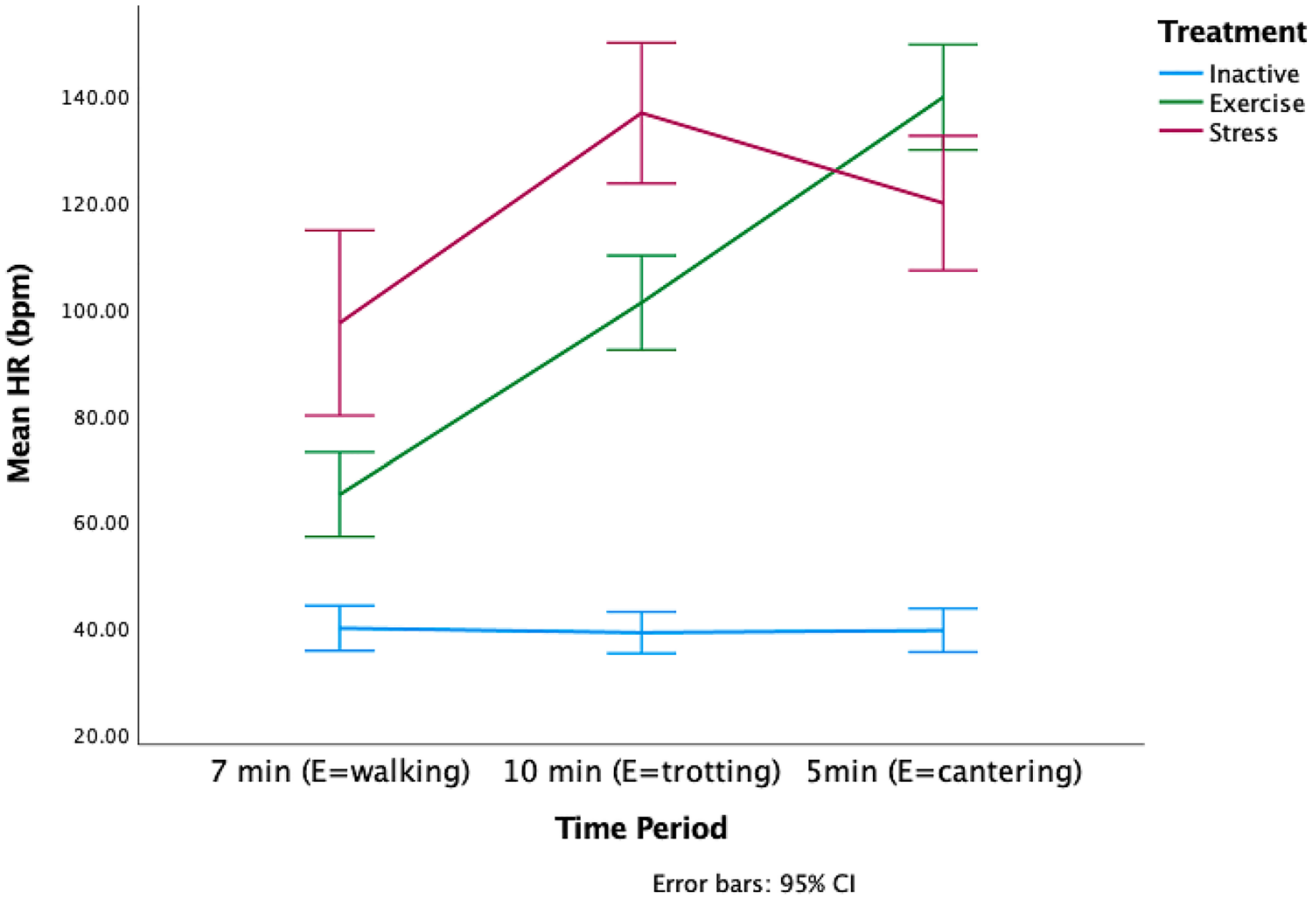
Change in mean HR during treatment across three time periods aligned to the exercise (E) horses’ workout of 7 min of walk, 10 min of trot and 5 min of canter. The I horses were inactive during the treatment and the speed and duration of activity of the S horses varied throughout the treatment.

The there was a significant difference in the HRs of each of the treatment groups during the treatment phase (GLMM: *F*_*8,112*_ = 65.97, *p* < 0.000001, Fig. 4). The GLMM model estimates and confidence intervals (Supplementary Table 2), indicated that the HRs of the E horses steadily increased across the treatment, whereas the HRs of the S horses decreased in the final five minutes. The I horses’ HRs did not vary.

### Salivary cortisol

**Figure 5:**
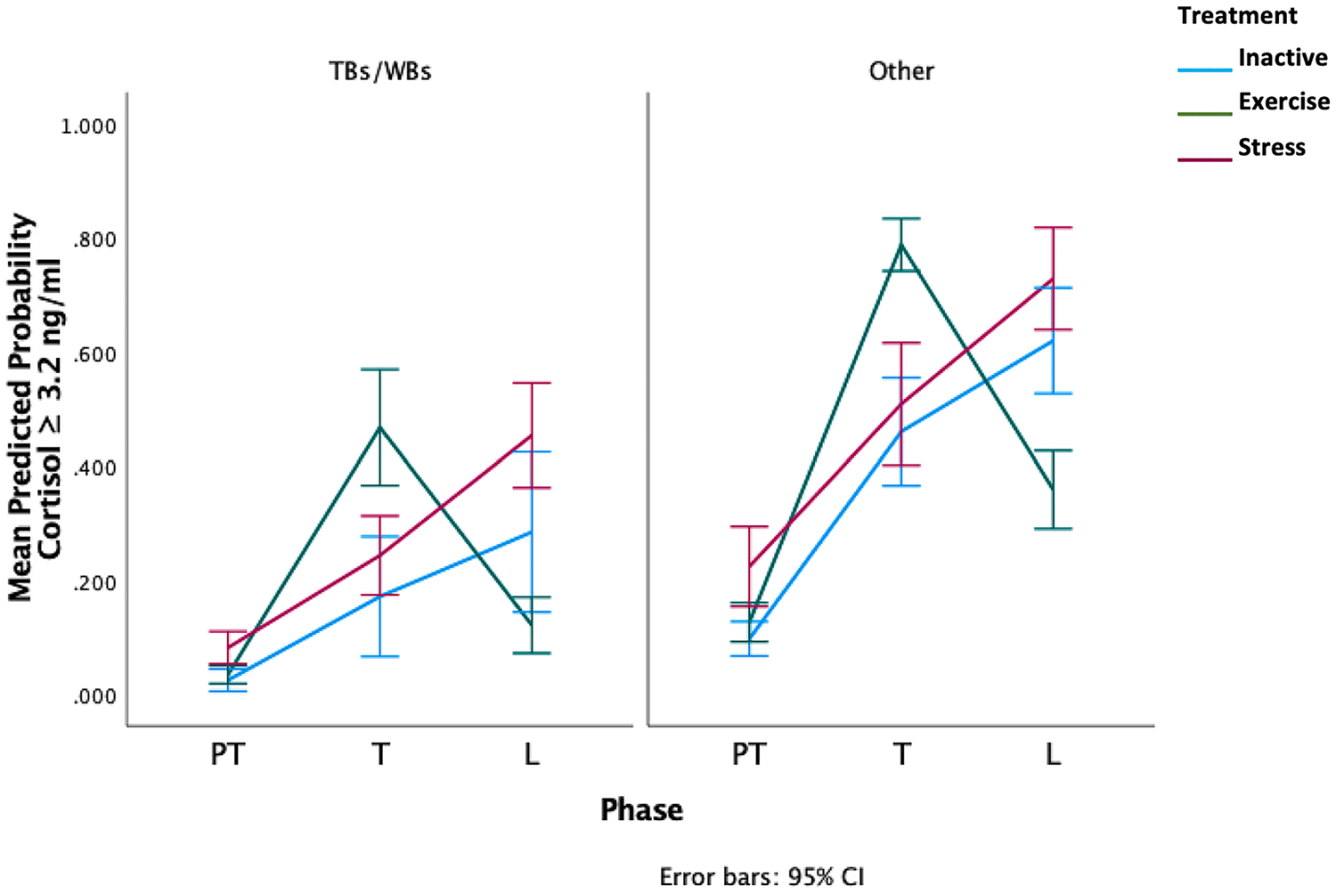
Predicted probability of high salivary cortisol (≥ 3.2 ng/ml) across the three phases. Values of < 0.5 have a lower probability of cortisol concentrations of 3.2 ng/ml or higher and values of 0.5 or greater have a higher probability of cortisol concentrations of 3.2 ng/ml or higher. TBs/WBs = Thoroughbred and warmblood breed horses, Other = Other breeds including Arab, Arab crosses, ponies, crossbreds. (Breed details can be found in Supplementary Table 1).

There was a significant treatment*phase interaction (*F*_8,113_ = 2.39, *p* = 0.02), with E horses more likely to have cortisol concentrations equal to or greater than 3.2 ng/ml after treatment and concentrations below 3.2 ng/ml after learning, whereas the and S horses were more likely to have higher concentrations after learning (Fig. 5). There was also a significant effect of breed as horses of thoroughbred and warmblood breeding (TB/WB) had a lower probability of cortisol values ≥ 3.2 ng/ml compared to other breeds (*F*_1,40_ =6.17, *p* = 0.014, Fig. 5, raw salivary cortisol means in Supplementary Table 3). Sampling time was not associated with raw cortisol values for any phase (Spearman’s rho: PT: *r*_*s*_ =—0.13, *p* = 0.4).*,* T: *r*_*s*_ = 0.02, *p* = 0.9, L: *r*_*s*_ =—0.09, *p* = 0.6).

### Spearman’s correlations-learning performance and physiological indicators

The number of trials to criterion was positively correlated with the raw cortisol values during learning (Spearman’s rho: *r*_*s*_ = 0.45, *p* = 0.004, Supplementary Fig. 2), but not with mean HR (*r*_*s*_ = −0.13, *p* = 0.42) or maximum HR (*r*_*s*_ = −0.07, *p* = 0.67).

## Discussion

This study provides novel evidence in horses that a short session of ridden exercise prior to learning may facilitate the acquisition of a negatively reinforced learning task, compared to inactivity or stress. The number of taps across treatment groups did not differ, suggesting that sensitivity to the whip stimulus did not influence acquisition of the task. The heart rates of the exercised and stressed horses were elevated during treatment compared to the inactive horses, and remained elevated during learning, however there was a difference in cortisol concentrations. The statistical model predicted that the exercised horses were more likely to have higher concentrations during the treatment than inactive and stress horses, but significantly lower concentrations during learning. Higher raw cortisol concentrations during learning were correlated with requiring more trials to reach the learning criterion. The putative neurobiological underpinnings of these results are discussed below.

Based on findings in other species, the more rapid acquisition of the learning task by the exercised horses in our study is likely to be mediated by increases in neurotransmitters such as noradrenaline and cortisol to concentrations at a dose eliciting beneficial effects on the prefrontal cortex-basal ganglia-amygdala neural network that facilitates instrumental learning^75^. The exercise treatment increased horses’ maximum HRs to 148 bpm which is likely to elicit noradrenaline release in circulation^65,76^ and consequently the brain^33^. Adrenaline and noradrenaline release is facilitated by the SAM axis which responds on a timescale of milliseconds to enable rapid responses to stressors, whether physiological such as exercise or psychosocial, such as stress^58^. Adrenergic molecules are believed to have a short half-life in the circulation^77^, however both Baragli et al.,^65^ and Hada et al.,^76^ reported blood concentrations took considerably longer to be normalised in horses at the conclusion of submaximal exercise and exposure to a novel object respectively. Noradrenaline concentrations in brain regions such as the amygdala and elsewhere can remain elevated for several hours following exercise or stress^78,79^. Noradrenaline enhances learning by rapidly increasing the firing rates of neurons via high affinity α2A adrenoreceptors that increase the strength of action potentials^80^ as well as via interactions with cortisol^81^.

In addition to the likely increase in noradrenaline, the exercise treatment also affected salivary cortisol concentrations during the treatment and subsequent learning. In comparison to the SAM axis, the HPA axis reacts to stress or exercise demands more slowly, on a timescale of between 10 and 30 min^11^. At 22 min, including 15 min of exercise at trot and canter carrying the weight of a rider, the exercise treatment was sufficient to increase the exercised horses’ cortisol concentrations compared to pre-test. However, at the conclusion of the learning phase which followed immediately after the treatment, their cortisol concentrations had fallen. Cortisol exerts rapid non genomic and delayed genomic effects, and the rapid effects are reported to occur within minutes of increases in concentration^75^. Cortisol rapidly binds to high affinity mineralocorticoid receptors in the hippocampus, facilitating glutamate release which in turn activates ionotropic receptors that regulate synaptic plasticity and the efficiency of synaptic transmission to support learning^82^. Slower genomic effects occur after a lag of around 20 min and consequently are unlikely to have influenced learning performance of the exercised horses, given the mean duration of their learning sessions was less than 20 min^47^. The memory enhancing effects of glucocorticoids and noradrenaline are dose dependent with moderate but not low or high concentrations being beneficial^83^ and consequently it is possible that exercise horses rapid acquisition of the learning task occurred under the influence of cortisol and noradrenaline at doses that facilitated learning in cortical and basal ganglia regions responsible for instrumental learning. This interpretation requires further research to confirm.

It is also possible that the negative reinforcement signals used by the riders during the exercise treatment may have facilitated the exercise horses’ acquisition of the negative reinforcement learning task. However, any such influence is likely to be small, given that all subjects were assessed as having a minimum level of training to enable them to be ridden and complete the demands of the exercise task prior to inclusion in the study, and all horses were handled using negative reinforcement techniques during the experiment. The riders were instructed to use the least amount of pressure to guide and control the horse during the exercise treatment and during the workout, the horses were retrieving memories of previously trained responses to the ridden cues, whereas the learning task involved the acquisition of new learning. The novelty of the task is demonstrated by the lack of a significant difference between the groups in the rate of learning (Supplementary Fig. 1) and the lack of significant differences in regards to the number of taps applied.

In comparison to the exercised horses, the stress horses’ learning was less efficient as they required significantly more trials to reach the criterion. The stress treatment involved a combination of exposure to objects that were inherently aversive, eliciting escape and avoidance responses involving physical activity associated with those responses. However, unlike the exercise treatment in which the duration of the workout was similar for all horses, and increased in energetic demand as the session progressed, there was variation in the duration and intensity of the physical activity of the stress horses, both within and between sessions. Consequently, the physiological responses to the stress treatment are likely to reflect the combination of the affective and physical aspects of the treatment. In common with the exercised horses, stressed horses’ maximum HRs during the treatment were sufficient to likely elicit noradrenaline release in the brain, however their cortisol concentrations were higher than exercised horses at the end of learning. During intense stress or fear conditioning which elicits strong glucocorticoid and noradrenaline release, the activity of the basolateral and central nuclei of the amygdala facilitate attention on the stressor at the expense of peripheral information^84^. This can result in a bias towards habitual or reflexive defensive behaviours via inhibition of corticostriatal control of goal-directed learning in favour of habit learning or species specific defensive behaviours that are mediated by the dorsolateral striatum and/or periaqueductal grey^85–88^. In humans this shift is a function of impaired goal-directed learning rather than a predominance of the habit learning, reflecting inhibition of flexible corticostriatal activity^89^. Smeets et al.,^90^ reported that mice with higher cortisol reactivity performed more habit-like responding in an appetitive task and reflexive freezing in rats during avoidance learning can inhibit the acquisition of some aversive instrumental tasks^91,92^. In the horse, reflexive-defensive locomotory behaviours can interfere with negative reinforcement learning^93^.

The stress treatment was designed to be of a duration and intensity mirroring some features of popular training methods that involve chasing the horse until it performs specific behaviours after which the chasing ceases^94^. In the context of this study, the stress treatment may have sensitised to the horse to the aversive characteristics of the learning task stimuli further increasing activity in the defensive neural network^95^, slowing their acquisition of the learning task. Kydd et al.,^96^ reported wide variability in the way this type of method is employed and that amateur trainers elicit more stress-like behaviours than professionals. The HRs reported here align with those reported in studies of professional trainers using this method^97,98^. High concentrations of glucocorticoids are associated with increased vigilance and activation of defensive circuits involving the amygdala, thalamus and periaqueductal grey that focus attention on the immediate threat at the expense of irrelevant details, which may have impaired the stress horse’s ability to focus on the details of the learning task^75,99^.

These data suggest that the learning performance of horses in an industry standard aversive NR task, is sensitive to exposure to stressors that combine affective and physical components as implemented here. Consequently, negative reinforcement learning may be impaired by training or handling methods that simultaneously cause substantial increases in cortisol and HRs to levels likely elicit strong noradrenaline release. Exposure to other stressors such as transport may lead to similar physiological responses to those reported here^30,70^ and consequently, it may be advantageous to avoid or delay training where horses have recently been exposed to situations which elicit these physiological responses.

In addition, uncontrollable stress can also impair acquisition of aversive instrumental tasks due to high concentrations of serotonin release from the dorsal raphe nucleus to prelimbic-dorsomedial-periaqueductal grey circuits, biasing inactivity and impairing escape learning^100^. The stress treatment in this study was designed to be uncontrollable. However, unlike in studies of uncontrollable stress in which subjects are restrained, limiting their abilities to trial behavioural responses to escape the stressor (for example in^50^), the horses here, could perform a range of behaviours during the treatment even though they did not facilitate escape from the stressor. The stress horses also performed escape responses to the aversive stimulus in the learning task so it is not clear if likely serotonin release arising from the uncontrollability of the stress treatment contributed to their slower acquisition of the task.

Irrespective of the treatment, horses with higher raw cortisol concentrations required the most trials to learn the task. It is possible that the learning task itself caused the increase in cortisol concentrations of the horses who required more trials to reach the criterion and consequently, a task-induced cortisol effect was responsible for the slower acquisition of the task in high cortisol horses, rather than extrinsic factors such as the stress or exercise treatments. Valenchon et al.,^20^ reported that participation in either a PR or NR learning task ± repeated short term stress exposures between blocks of trials increased salivary cortisol concentrations relative to basal, however only the PR + stress group showed a significantly larger increase compared to the NR ± stress and PR- stress groups. However, in the case of our study, with the exception of the inactive horses, the stress and exercise horses commenced learning under the influence of treatment affected physiology and consequently it not possible to parse the effects of learning related effects on cortisol or HR separately from the effects of the two active treatments. Valenchon et al.,^21^ reported that salivary cortisol concentrations of horses exposed to a 30 min stressor prior to positive reinforcement learning peaked at the conclusion of the learning session. Their horses’ learning performance was transiently enhanced (early trials) however the overall learning performance did not differ from controls. In comparison, in this study, higher cortisol concentrations were associated with reduced learning efficiency and fewer correct responses were made by all horses in the early learning trials (Supplementary Fig. 1).

The inactive treatment was not expected to elicit increases in salivary cortisol concentrations or HR (and by proxy noradrenaline). However, the inactive horses’ cortisol concentrations at the conclusion of the were unexpectedly high with a similar trajectory to the stress horses who were physically active as well as exposed to the stressor. Despite the presence of the companion horse throughout the pre-test and treatment phases, at the conclusion of the combined phases, the horses had been restrained for 35 min. The resulting restriction of their behavioural choices and their inability to remove themselves from the restrictions of the restraint may be been experienced as a stressor^101^. Consequently, this aspect of the treatment, in combination with a learning task that exposed them to aversive stimuli which they were slower to learn, may have contributed to their high cortisol concentrations at the end of the learning session. However, at no time during the experiment did the inactive horses HRs increase to levels associated with systemic noradrenaline release. The facilitating effects of cortisol on learning require simultaneous adrenergic activity^35^, and consequently it may be that the inactive horses did not benefit from their higher cortisol concentrations due to the lack of accompanying noradrenaline release. There are currently no data to determine whether stress exposure that elicits low to moderate HR increases in horses, also influences brain concentrations of noradrenaline. The data presented here suggests that in the absence of an obvious concurrent increase in noradrenaline, the high cortisol concentrations impaired learning in the inactive horses, as has been reported in other species^81^.

There was a significant breed effect on the probability of high cortisol concentrations, during the T and L, with non-TB/WB horses having a higher probability of high cortisol than the TB/WB horses. In common with the other breeds used in the study, the TB/WB horses were a mixture of horses that were either in active training for eventing competitions or horses that had been in low to moderate exercise for at least the month prior to the experiment. In comparison to our data, Sauer et al.,^102^ reported that warmblood and thoroughbred sport horses had higher salivary cortisol concentrations in response to ACTH challenge than French-Montagne breed horses. Cortisol responsive phenotypes have been identified in Japanese quail^103^, mice^104^ and cortisol reactivity in humans is associated with some psychiatric disorders^105^. Further research could explore whether similar phenotypes also exist in horses and whether this influences their cognitive performance in NR learning.

BDNF, a neurotrophin associated with enhanced learning^106^, and dopamine^107^ are also potential candidates mediating the relative differences in learning acquisition between the treatment groups, however in the absence of the BDNF analysis in particular, this requires confirmation. Spontaneous eyeblink rate, a validated marker of dopaminergic activity in basal ganglia regions involved in instrumental learning, has been correlated with salivary cortisol concentrations in horses exposed to a short term stressor^3^ and this combined with the use of circulating BDNF measures as has been used in the human exercise and cognition literature^108^ could provide a mechanism to explore these issues.

We note the inferential nature of the analysis of these results and the low precision offered by relying on peripheral concentrations or proxies of neurotransmitter release and activity in neural networks associated with instrumental learning. The limitations of this approach have been identified in relation to studies of human cognition that rely on similar measures^17,61^. In the absence of the opportunities for assessing brain activity in real time in horses that are available for rodent models, this inferential approach provides a mechanism to explore the putative neurobiological underpinnings of behaviour in equine subjects. It is to be hoped that new tools to improve the robustness and precision of methods to analyse equine behaviour and neurobiology in relation to cognition and affect will be developed in the future.

## Conclusion

This results of this preliminary study suggest that exercise of moderate intensity and low cognitive load may have a beneficial effect on negative reinforcement learning in horses. The learning task mirrored industry conditions and consequently this finding has direct relevance to industry. In the absence of data obtained directly equine brain regions, findings from other species suggest that the cognitive benefits of the exercise in this study were likely to be mediated by beneficial increases in cortisol and noradrenaline interacting with dopamine, BDNF and other neurotransmitters that collectively enhance synaptic plasticity and excitability in regions associated with learning. In comparison the stress exposure impaired learning, possibly due to a glucocorticoid and noradrenaline mediated upregulation of salience and inflexible learning networks at the expense of activity in flexible networks that facilitate learning novel tasks. Training activities conducted while the horse is under the influence of high cortisol concentrations, whether from extrinsic or training related stressors, appear to impair aversive instrumental learning acquisition compared to lower cortisol concentrations. This study supports industry advice that a short period of physical warm-up of low cognitive demand, with the horse moving freely and calmly may set the horse up for enhanced learning during a training session, particularly when compared to commencing training without any prior physical activity. In contrast, exposure to uncontrollable stress prior to a training session should be avoided as learning may be impaired.

## Materials and methods

The methods detailed below conform to ARRIVE guidelines.

### Horses

A convenience sample of riding horses were sourced from private owners (*n* = 41; 17 mares, 1 stallion, 23 geldings, age range 3 to 20 years, mean 8.5 ± 5.1 years-breed details Supplementary Table 1). All horses had a minimum level of training such that they could be ridden a walk, trot and canter on a loose rein. Prior to recruitment, they were given two taps on the gluteal area of the hindquarters level with the hip joint with 1.1 m long dressage whip and only horses that did not make any locomotory reaction to the whip were included in the study. The experiment was conducted at three separate professional horse training establishments in rural NSW, Australia.

Horses at locations 1 and 2 resided at the venue, whereas horses at location 3 were a mix of resident (*n* = 5) and externally owned animals (*n* = 12). The externally owned animals were transported to the venue 14 days prior to testing. During this period the external horses’ suitability for potential allocation to the exercise treatment was assessed via two or three ridden sessions on the arena in which the testing was to take place to facilitate habituation to the arena and the rider. One horse that was not sufficiently educated to be safely ridden at the level required for the exercise treatment was excluded from the study. All horses were housed in either shared or single paddocks with visual contact with other horses during the experiment and for at least the previous month while at their home locations. They were fed a pasture/hay based diet with supplementary concentrates at location 2, according to the requirements of the owner/managers.

### Ethical approval

The use of horses in this project was authorised by the Charles Sturt University Animal Care and Ethics Committee (Approval Authority 18,028) and all methods were performed in accordance with the relevant guidelines and regulations as per the conditions of this approval.

### Experimental procedure

Horses were semi randomised into one of the treatment groups: inactive (I, *n* = 14), exercise (E, *n* = 13), and stress (S, *n* = 14), dependent on the operational needs of the owner/managers at each location. Details of the breed, sex and treatment allocation for each location can be found in Supplementary Table 1. Four horses were tested per day over a three day period at locations 1 and 2 and a five day period at location 3. The order of testing was determined by the operational needs of each facility and for the majority of testing days, included at least one horse from each treatment group each day. At the commencement of data collection, the E horses were fitted with their usual dressage or jumping saddle and snaffle bridle and the C and S horses wore an elasticised surcingle, halter and lead rope. A heart rate monitor set to ‘R-R’ mode (HRM-Polar V800 receiver, Polar H10 sensor and Polar Equine Electrode base, Polar Electro Oy, Finland) was fitted under the saddle or surcingle.

There were three phases: pre-test (PT), treatment (T), and learning (L) which were conducted in succession without a break. Horses were tested individually and underwent all three phases before the next horse was tested. The PT phase took place in the company of another horse nearby and the during the T phase, I horses remained in close proximity to a companion and the S and E horses underwent the treatment on their own. The L phase was conducted adjacent to the PT location and in visual contact with other horses paddocked near by. At the conclusion of the testing, the horses were returned to their respective paddocks. At each facility, the preparation area, exercise area and stress area were located between 50 and 100 m from the learning location and consequently, the time taken to travel between the test areas was between 20 and 90 s. No physiological data was collected during movement between test areas.

### Pre-test phase

The horse was tied to a railing in the presence of a familiar companion horse and left undisturbed for 15 min after which the saliva and blood samples were collected. Horses in the S and E treatment groups were then led to the relevant location and I horses were left with the companion horse in the same area.

### Treatment phase

#### Exercise treatment

The horses were ridden by competent riders who were familiar with the horse at each location, including the rider who undertook the familiarisation rides of externally owned horses at location 3. The freestyle workout in a natural outline on a loose rein involved seven minutes of walking, followed by 10 min of trotting and concluded with five minutes of cantering. The riders were instructed to include direction changes during the workout and only use as much rein and leg pressure as needed to steer the horse and maintain the correct gait whilst not influencing the horse’s head carriage or other locomotory characteristics.

#### Stress treatment

The horse was released into a round yard, (RY-diameter range 15–22 m, 1.8 m height, dirt or sand surfaced) and left undisturbed for two minutes. Thereafter the stress items were introduced into the round yard and the stress exposure commenced. The stress items were chosen for each location based on the size of the RY to ensure the horse could not escape or minimise their exposure to the stressors whilst minimising the risk of injury to the horse, with the same 1.5 m rubber ball used at all locations: Location 1: (RY = 22 m diameter) 1.5 m diameter rubber ball and 2mx3m plastic tarpaulin, Location 2:(RY = 15 m diameter) 1.5 m rubber ball, Location 3: (RY = 20 m diameter), 2 × rubber balls 1.5 m and 75 cm diameter. The ball(s) were pushed, bounced, tapped and tossed and the tarp moved in an unpredictable manner so that the horse could not escape or control their exposure to the stimuli. The intensity of the manipulation was adapted to the responses of the horse to minimise risks of injury. If the horse showed signs of habituation towards the items (such as stepping towards or decreasing alertness), the experimenter increased the activity of the items until the horse responded by moving away. Where the horse exhibited strong flight behaviour or other behavioural indicators of strong fear, the operator reduced the speed and proximity of the items relative to the horse, however care was taken to ensure that no specific behavioural response to the stressor was reinforced. The stressor was applied continuously for 20 min.

#### Inactive treatment

The horse was tethered to a railing and left undisturbed for 22 min in the company of the companion horse (tethered or yarded no greater than 1 m from the test horse).

### Learning phase (L)

The learning location was a flat area adjacent to the pre-test area marked with orange traffic cones spaced 12 m apart. The same person (CH) undertook the learning task procedure for all locations. The trainer stood on the right side of the horse with the reins or lead rope held in the right hand and the 1.10 m long dressage whip was held in the left hand. The whip was firstly raised to the tap position, level with the hip joint. If the horse did not respond within 2 s, gentle tapping with a light pressure was commenced. The taps were applied at the same steady rhythm and low intensity and only to the right side of the hindquarters as per Fig. 1. The tapping was maintained until the horse made an attempt to move the hindquarters towards the left (away from the whip). The tapping ceased immediately the horse lifted a hind leg towards the midline, even if the movement was small. Trials were conducted in blocks of five, with a 10 s break between blocks. Each response was graded by the trainer according to a predetermined scoring system. The trainer called the performance score to a helper who recorded the score in a notebook. Responses were scored from 1 to 3, with a score of 1 denoting no or one sideways step, 2 denoting two sideways steps and 3 denoting three or more sideways steps. The duration of the session was also recorded. The learning criterion was set at three consecutive responses at score 2, from two or less whip taps. When the horse reached the learning criterion the phase was concluded.

### Physiological sampling and analysis

Saliva samples were collected at the conclusion of each phase in Salivette synthetic rolls (Sarstdet, Nümbrecht, Germany) held inside the horse’s mouth for 1 min on artery forceps. The sample was placed on ice and later frozen at −20 °C at the conclusion of each day’s testing and maintained at this temperature until analysis. Blood samples for BDNF assay were collected after the PT and L phases via venepuncture but were not able to be analysed due to a university laboratory freezer malfunction after collection. The HRM was paused during the collection of samples and reset at the beginning of each phase.

Saiva samples were thawed in the original collection tube, centrifuged at 1500 g for 10 min and aliquoted into 1.5 ml Eppendorf™ tubes after which they were assayed in an undiluted form according to the kit manufacturer’s directions (Salimetrics Salivary Cortisol Assay, State College, Pennsylvania) . The optical density of the plates was read at 450 nm with correction filter at 490 nm (BioTek ELX 800, Winooski, Vermont). A standard curve was calculated using four parameter logistic regression. The cortisol concentration of each well was calculated in ng/ml. All samples, standards and controls were assayed in duplicate and the mean and coefficient of variation (COV) calculated. The mean intra plate COV was 5% and the inter plate COV was 15%.

### Statistical analysis and data handling

The HR data stored in the HRM receiver were transferred to the Polar Flow web interface (www.polarflow.com) via the Polar Flow phone app and these files were uploaded to Kubios (Kubios HRV Premium, ver. 3.2.0, Kubios Oy, Kuopio, Eastern Finland). The raw R-R data were corrected using the Kubios automatic artefact correction mode^109^ and the data subsequently exported to MS Excel.

The statistical analysis was undertaken using SPSS (IBM, Armonk NY, Release 27) and model testing of cortisol data was performed using R (RStudio team, 2020). The learning related variables were not normally distributed (number of trials to reach criterion, rate of learning, and the number of taps applied-Shapiro–Wilk *p* < 0.05) and were analysed with a Kruskal–Wallis-Independent samples test (KW) with the relevant learning related variable as dependent variable and treatment or location as independent variables. The rate of learning (number of correct responses per five trials) was determined by calculating the proportion of correct responses per five trials (two step response) which were plotted on individual scatter graphs for each horse from which the slope of least squares regression equation was calculated. Rate of learning was then compared between treatments with a KW (data in Supplementary Materials). The SPSS Bonferroni’s adjustment method^110^ was applied during post hoc pairwise comparisons to correct for multiple comparisons for KW tests. Data from the first 60 trials for each horse were analysed as only two horses required more than 60 trials to reach the criterion (65 and 110 trials).

Heart rate data were parametric and the mean, minimum and maximum HRs were analysed with a General Linear Model with Repeated Measures (GLM-RM) with phase as the repeated measure, HR factor as the dependent variable and treatment or location as fixed effects. The majority of the data were not spherical (Mauchly’s test of sphericity) and Greenhouse-Geissler adjusted degrees of freedom with decimal places are reported where relevant. Individual phases were analysed with a univariate GLM with HR factor as the dependent variable and post hoc comparisons carried out with Tukey’s HSD correction. The Treatment HR traces were split into three blocks based on the duration of the E treatment (seven minutes walking, 10 min trotting and five minutes cantering) and were analysed with a Generalised Linear Mixed Model (GLMM) with mean HR as the target, treatment*time period as fixed effect and horse as random effect. The cortisol data were binomially distributed and not amenable to normalisation. The data were converted to a dichotomous variable based on Jenks natural breaks classification method^111^ which split the data on the value of 3.2 ng of cortisol per ml of saliva. Horses with values lower than 3.2 ng/ml were coded as “0” (low) and those with values of 3.2 ng/ml or higher were coded “1” (high). Horse breed was converted to a dichotomous variable, with warmbloods and thoroughbreds (54%) coded as 0 and other breeds (46%) coded as 1. All possible GLMM models were fitted with binary logistic regression link function using R. Models were ranked using an information theoretic approach based on Akaike’s Information Criteria (AIC^112^). The most suitable model included phase*treatment with breed code as a fixed effect (AIC :138.2599, R^2^ 0.512, %correctly predicted: 87.1). This model was then analysed in SPSS with a GLMM binary logistic regression with binary cortisol code as the target and treatment*phase and breed as fixed effects with horse as random effect. The predicted probabilities of high cortisol ($\ge$ 3.2 ng/ml) in any given phase or treatment were generated, with values < 0.5 indicating a reduced probability of high cortisol and values > 0.5 indicating an increased probability of high cortisol.

Spearman’s rank order correlations were conducted to determine if there were significant relationships between raw cortisol values and sampling times for each phase as well as between learning performance, HR and raw cortisol values. Data are reported as mean + 95% confidence intervals. Statistical significance was set at *p* < 0.05.

## Supplementary Information

Supplementary Information.
