# Author Correction: The effect of stress and exercise on the learning performance of horses

**DOI:** 10.1038/s41598-022-09569-z

**Published:** 2022-03-29

**Authors:** Cathrynne Henshall, Hayley Randle, Nidhish Francis, Rafael Freire

**Affiliations:** School of Agricultural, Environmental and Veterinary Sciences, Charles Sturt University, Wagga, Wagga, NSW Australia

Correction to: *Scientific Reports* 10.1038/s41598-021-03582-4, published online 04 February 2022

The Supplementary Information file published with this Article contained a repeated error, where the term “inactive” was incorrectly given as “control.” The original Supplementary Information file is provided below.

This error has now been corrected in the Supplementary Information file that accompanies the original Article.

## Supplementary Information

Supplementary Information.

