# Supplementary material for: The effect of stress and exercise on the learning performance of horses
Source: Sci Rep. 2022 Feb 4;12:1918. doi: 10.1038/s41598-021-03582-4 (PMC8816904; doi:10.1038/s41598-021-03582-4)
Supplement: Supplementary file 1 — Supplementary Information. [file 41598_2021_3582_MOESM1_ESM.pdf]

# Supplementary Materials

***The effect of stress and exercise on the learning performance of horses.***

Cathrynne Henshall*, Hayley Randle, Nidhish Francis, Rafael Freire

School of Agricultural, Environmental and Veterinary Sciences

Charles Sturt University, Wagga Wagga, NSW Australia

*chenshall@csu.edu.au

*Supplementary Table 1- Details of horses used in the experiment*

| Breed | Total | Cortisol breed code | Sex | | | Group | | | Location | | |
|---|---|---|---|---|---|---|---|---|---|---|---|
| | | | **M** | **G** | **S** | **I** | **E** | **S** | **1** | **2** | **3** |
| Thoroughbred | 12 | WB/TB | 3 | 9 | | 2 | 4 | 5 | 1 | 2 | 9 |
| Warmblood | 9 | WB/TB | 6 | 2 | 1 | 3 | 3 | 3 | | 9 | |
| Arab/Arab crossbreds | 5 | Other | 2 | 3 | | 2 | 1 | 2 | 2 | | 3 |
| Pony breeds | 4 | Other | 2 | 2 | | 3 | 1 | 2 | 2 | | 2 |
| Unknown breeding | 4 | Other | 2 | 2 | | 1 | 1 | 1 | 2 | | 2 |
| Quarter horse/paint bred | 3 | Other | | 3 | | 2 | | 1 | 1 | 1 | 1 |
| Draught breed crossbred | 2 | Other | | 2 | | | 2 | | | | 2 |
| Andalusian x Standardbred | 2 | Other | 2 | | | 1 | 1 | | 2 | | |

*Supplementary Table 1. Summary of breed and sex characteristics. Key: M=mare, G=gelding, S=stallion, I=Inactive, E=exercise, S=stress, WB/TB= warmblood or thoroughbred. Cortisol breed code= breeds assigned to each code for GLMM-BLR model.*

## *Duration of learning and taps applied during learning*

E horses reached the learning criterion more quickly than I but not S horses and there was no significant difference between I and S (Mean ± SD: I: 509.83 ±276.23s E: 308.00s ±163.47s, S:497.70 ±333.97s, ($\chi^2$ *(2)*= 6.307, *p*=0.043). Pairwise comparisons with SPSS Bonferroni correction: E-S: ($\chi^2$ *(2 )*= -9.346, *p*=0.128, E-I: ($\chi^2$ *(2)*=10.703,*p*=0.061, S-I: ($\chi^2$ *(2)*= 1.357, *p*=1.00). The learning duration did not differ between the experiment locations ($\chi^2$

*(2)=* 0.847, *p*=0.655) and nor did the mean number of taps applied during learning ($\chi^2$ *(2)=* 1.074, *p*=0.584)

*Rate of learning- statistical analysis and results*

The rate of learning was determined by calculating the proportion of correct responses per five trials for each horse and then generating a scatter plot from which slopes of least squares regression lines were calculated.  These data were then compared with a KW, followed by post hoc pairwise comparisons with SPSS Bonferroni's correction to correct for multiple comparisons.

The rate of learning approached significance between the groups ($\chi^2$ *(2)=* 5.986, *p*=0.05). However pairwise comparisons with SPSS Bonferroni correction revealed no significant differences (E-I horses ($\chi^2$ *(2)*=--10.326 *p*=.061), E-S horses ($\chi^2$ *(2)=* 8.385, *p*=0.218, S-I horses ($\chi^2$ *(2)=* -1.962, *p*=1.00).  During the first three blocks of 5 trials (15 trials), the percentage of correct responses (2 response) were similar across the groups (Supplementary Fig. 2). However, as the session progressed, the exercise horses performed an increasing percentage of correct responses compared to the inactive and stress horses.  There was no difference in the rate of learning across the three locations ($X^2$ *(2)=* 3.066, *p*=0.216).

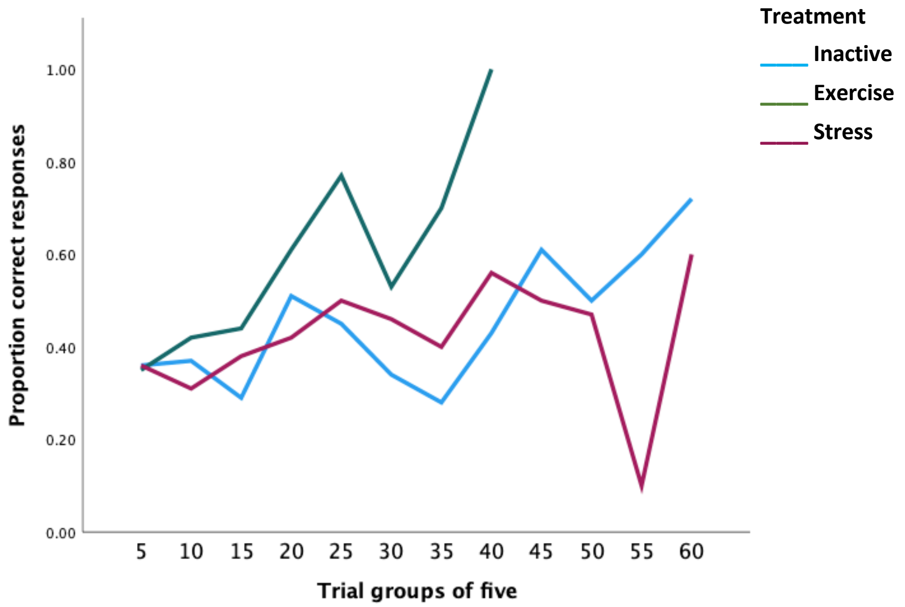


*Supplementary Figure 1- Percentage of correct responses (2 consecutive responses) per block of five trials.*

*Minimum and Maximum heart rate data*

During PT, the minimum and maximum HRs (bpm) were similar for all groups (Minimum HR: $F_{2,38}$ =0.028,*p*=0.973, Maximum HR: $F_{2,38}$ =1.826,*p*=0.175). Minimum HRs during T differed between the I and S horse but not the E horses, (Mean difference: I-E:-8.91[95% CI -19.70-1.88], *p*=0.123, I-S:-13.77 [95%CI -24.35- -3.16],*p*=0.008, E-S:-4.85 [95%CI -15.64-5.94], *p*=0.523). During L, the minimum HRs of the I horses were lower than the E or S horses (Mean difference: I-E:-12.39 [95% CI -20.52- -4.27], *p*=0.002, I-S:-15.50 [95% CI -23.48- -7.52], *p*=<0.001, E-S:-3.11 [95% CI -11.23-5.02], *p*=0.624).

The maximum HRs of the E and S horses were significantly higher than the I horses during T, (Mean difference-I-E:-75.32 [-111.84- -31.81 95% CI], *p*<0.001,I-S: -92.13 [-134-83- -49.43 95% CI], *p*=<0.001, E-S:-16.81 [-60.32-26.71 95% CI], *p*=0.617), whereas during L, only the S horses' maximums were higher than the I horses, (Mean difference-(I-E:-34.71 [-71.62-2.19 95% CI], *p*=0.069,I-S:-57.05 [-93.27- -20.83 95% CI], *p*=0.001, E-S:-22.34 [-57.25-14.57 95% CI], *p*=0.314).

*Supplementary Table 2- Generalised linear mixed model generated estimated means of HRs during Treatment.*

| Treatment | Time Period | Mean | 95% Confidence Interval | |
|---|---|---|---|---|
| | | | Lower | Upper |
| Inactive | Walk 7 min | 56.227 | 43.309 | 69.144 |
| | Trot 10 min | 55.818 | 42.901 | 68.736 |
| | Canter 5min | 54.583 | 41.666 | 67.500 |
| Exercise | Walk 7 min | 84.993 | 71.588 | 98.398 |
| | Trot 10 min | 119.569 | 106.164 | 132.974 |
| | Canter 5min | 155.048 | 141.127 | 168.969 |
| Stress | Walk 7 min | 172.137 | 159.219 | 185.054 |
| | Trot 10 min | 189.177 | 176.260 | 202.095 |
| | Canter 5min | 157.128 | 143.751 | 170.505 |

*Supplementary Table 2-GLMM estimated means of HRs during three time periods in the Treatment phase and 95% confidence intervals. Time periods are aligned to the Exercise treatment ridden workout including duration and gait for each phase of the treatment.*

*Supplementary Table 3-Mean cortisol concentrations*

| Phase | Treatment group | Mean (ng/ml) | 95% Confidence Interval | |
|---|---|---|---|---|
| | | | Lower | Upper |
| Pre-test | Inactive | 1.184 | .110 | 2.259 |
| | Exercise | 1.182 | -.0246 | 2.389 |
| | Stress | 1.681 | .3287 | 3.038 |
| Treatment | Inactive | 3.196 | 1.195 | 5.198 |
| | Exercise | 4.875 | 2.876 | 6.874 |
| | Stress | 3.351 | 1.529 | 5.172 |
| Learning | Inactive | 4.426 | 2.585 | 6.268 |
| | Exercise | 2.202 | .758 | 3.647 |
| | Stress | 3.916 | 2.226 | 5.607 |

*Supplementary Table 3- Mean raw cortisol measurements in ng/ml for each treatment group and phase.*

*Supplementary Figure 2- Scatterplot of ranked trials to criterion versus ranked cortisol concentrations during learning.*

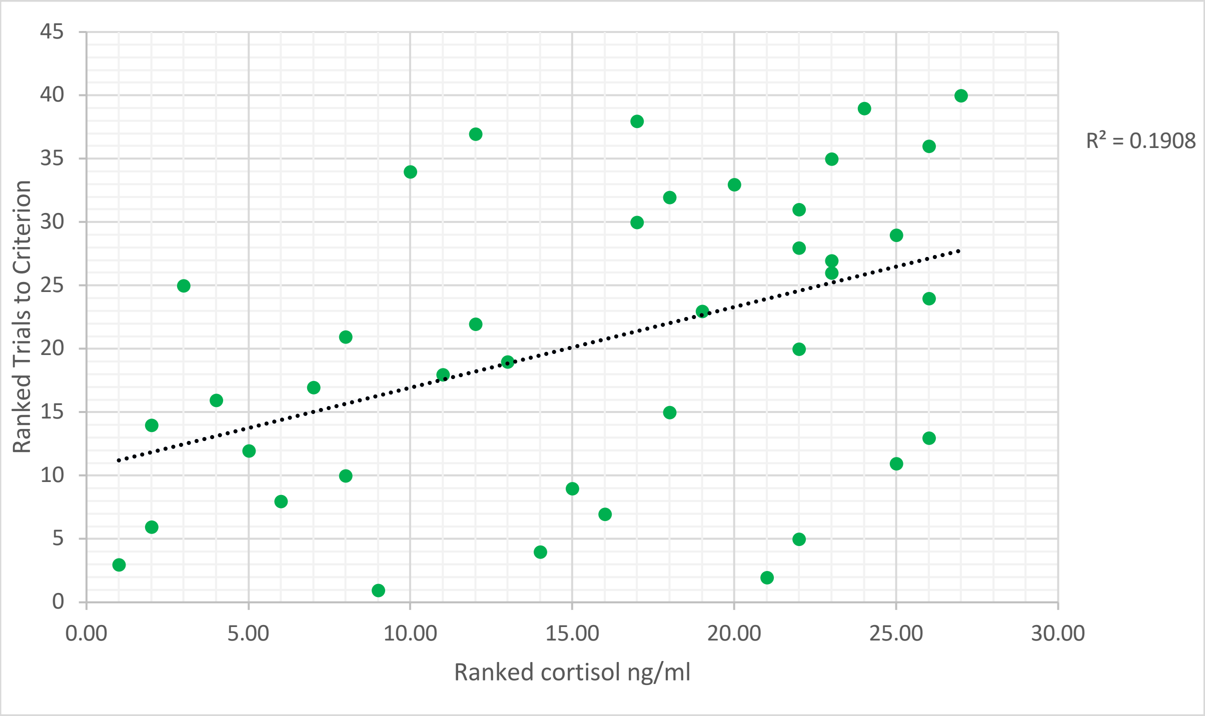


*Supplementary Figure 2- Scatterplot of ranked trials to criterion v ranked cortisol concentrations during learning.*
