# Supplementary material for: Author Correction: The effect of stress and exercise on the learning performance of horses
Source: Sci Rep. 2022 Mar 29;12:5322. doi: 10.1038/s41598-022-09569-z (PMC8964674; doi:10.1038/s41598-022-09569-z)
Supplement: Supplementary file 1 — Supplementary Information. [file 41598_2022_9569_MOESM1_ESM.pdf]

Supplementary Materials

The effect of stress and exercise on the learning performance of horses.

Cathrynne Henshall*, Hayley Randle, Nidhish Francis, Rafael Freire

School of Agricultural, Environmental and Veterinary Sciences

Charles Sturt University, Wagga Wagga, NSW Australia

*chenshall@csu.edu.au

Supplementary Table 1- Details of horses used in the experiment

		Cortisol breed code	Sex			Group			Location		
**Breed**	**Total**		**M**	**G**	**S**	**C**	**E**	**S**	**1**	**2**	**3**
Thoroughbred	12	WB/TB	3	9		2	4	5	1	2	9
Warmblood	9	WB/TB	6	2	1	3	3	3		9	
Arab/Arab crossbreds	5	Other	2	3		2	1	2	2		3
Pony breeds	4	Other	2	2		3	1	2	2		2
Unknown breeding	4	Other	2	2		1	1	1	2		2
Quarter horse/paint bred	3	Other		3		2		1	1	1	1
Draught breed crossbred	2	Other		2			2				2
Andalusian x Standardbred	2	Other	2			1	1		2		

Supplementary Table 1. Summary of breed and sex characteristics. Key: M=mare, G=gelding, S=stallion, C=control, E=exercise, S=stress, WB/TB= warmblood or thoroughbred. Cortisol breed code= breeds assigned to each code for GLMM-BLR model.

Duration of learning and taps applied during learning

E horses reached the learning criterion more quickly than C but not S horses and there was no significant difference between C and S (Mean ± SD: C: 509.83 ±276.23s E: 308.00s ±163.47s, S:497.70 ±333.97s, (χ^2 *(2)*= 6.307, *p*=0.043). Pairwise comparisons with SPSS Bonferroni correction: E-S: (χ^2 *(2)*= -9.346, *p*=0.128, E-C: (χ^2 *(2)*=10.703,*p*=0.061, S-C: (χ^2 *(2)*= 1.357, *p*=1.00). The learning duration did not differ between the experiment locations (χ^2

(2)= 0.847, *p*=0.655) and nor did the mean number of taps applied during learning (χ^2 *(2)=* 1.074, *p*=0.584)

Rate of learning- statistical analysis and results

The rate of learning was determined by calculating the proportion of correct responses per five trials for each horse and then generating a scatter plot from which slopes of least squares regression lines were calculated. These data were then compared with a KW, followed by post hoc pairwise comparisons with SPSS Bonferroni's correction to correct for multiple comparisons.

The rate of learning approached significance between the groups (χ^2 *(2)=* 5.986, *p*=0.05). However pairwise comparisons with SPSS Bonferroni correction revealed no significant differences (E-C horses (χ^2 *(2)=*--10.326 *p*=.061), E-S horses (χ^2 *(2)=* 8.385, *p*=0.218, S-C horses (χ^2 *(2)=* -1.962, *p*=1.00). During the first three blocks of 5 trials (15 trials), the percentage of correct responses (2 response) were similar across the groups (Supplementary Fig. 2). However, as the session progressed, the exercise horses performed an increasing percentage of correct responses compared to the control and stress horses. There was no difference in the rate of learning across the three locations (X^2 *(2)=* 3.066, *p*=0.216).

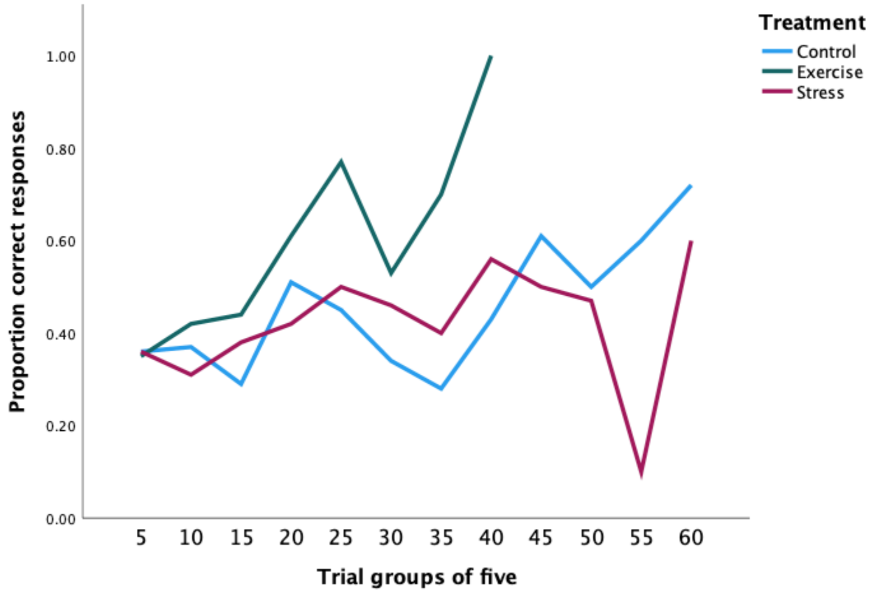

Supplementary Figure 1- Percentage of correct responses (2 consecutive responses) per block of five trials.

Minimum and Maximum heart rate data

During PT, the minimum and maximum HRs (bpm) were similar for all groups (Minimum HR: $F_{2,38}$ =0.028,*p*=0.973, Maximum HR: $F_{2,38}$ =1.826,*p*=0.175). Minimum HRs during T differed between the C and S horse but not the E horses, (Mean difference: C-E:-8.91[95% CI -19.70-1.88], *p*=0.123, C-S:-13.77 [95%CI -24.35- -3.16],*p*=0.008, E-S:-4.85 [95%CI -15.64-5.94], *p*=0.523). During L, the minimum HRs of the C horses were lower than the E or S horses (Mean difference: C-E:-12.39 [95% CI -20.52- -4.27], *p*=0.002, C-S:-15.50 [95% CI -23.48- -7.52], *p*=<0.001, E-S:-3.11 [95% CI -11.23-5.02], *p*=0.624).

The maximum HRs of the E and S horses were significantly higher than the C horses during T, (Mean difference-C-E:-75.32 [-111.84- -31.81 95% CI], *p*<0.001,C-S: -92.13 [-134-83- -49.43 95% CI]', *p*=<0.001, E-S:-16.81 [-60.32-26.71 95% CI], *p*=0.617), whereas during L, only the S horses' maximums were higher than the C horses, (Mean difference-(C-E:-34.71 [-71.62-2.19 95% CI], *p*=0.069,C-S:-57.05 [-93.27- -20.83 95% CI], *p*=0.001' E-S:-22.34 [-57.25-14.57 95% CI], *p*=0.314).

Supplementary Table 2- Generalised linear mixed model generated estimated means of HRs during Treatment.

Treatment	Time Period	Mean	95% Confidence Interval	
			Lower	Upper
Inactive	Walk 7 min	56.227	43.309	69.144
	Trot 10 min	55.818	42.901	68.736
	Canter 5min	54.583	41.666	67.500
Exercise	Walk 7 min	84.993	71.588	98.398
	Trot 10 min	119.569	106.164	132.974
	Canter 5min	155.048	141.127	168.969
Stress	Walk 7 min	172.137	159.219	185.054
	Trot 10 min	189.177	176.260	202.095
	Canter 5min	157.128	143.751	170.505

Supplementary Table 2-GLMM estimated means of HRs during three time periods in the Treatment phase and 95% confidence intervals. Time periods are aligned to the Exercise treatment ridden workout including duration and gait for each phase of the treatment.

Supplementary Table 3-Mean cortisol concentrations

Phase	Treatment group	Mean (ng/ml)	95% Confidence Interval	
			Lower	Upper
Pre-test	Control	1.184	.110	2.259
	Exercise	1.182	-.0246	2.389
	Stress	1.681	.3287	3.038
Treatment	Control	3.196	1.195	5.198
	Exercise	4.875	2.876	6.874
	Stress	3.351	1.529	5.172
Learning	Control	4.426	2.585	6.268
	Exercise	2.202	.758	3.647
	Stress	3.916	2.226	5.607

Supplementary Table 3- Mean raw cortisol measurements in ng/ml for each treatment group and phase.

Supplementary Figure 2- Scatterplot of ranked trials to criterion versus ranked cortisol concentrations during learning.

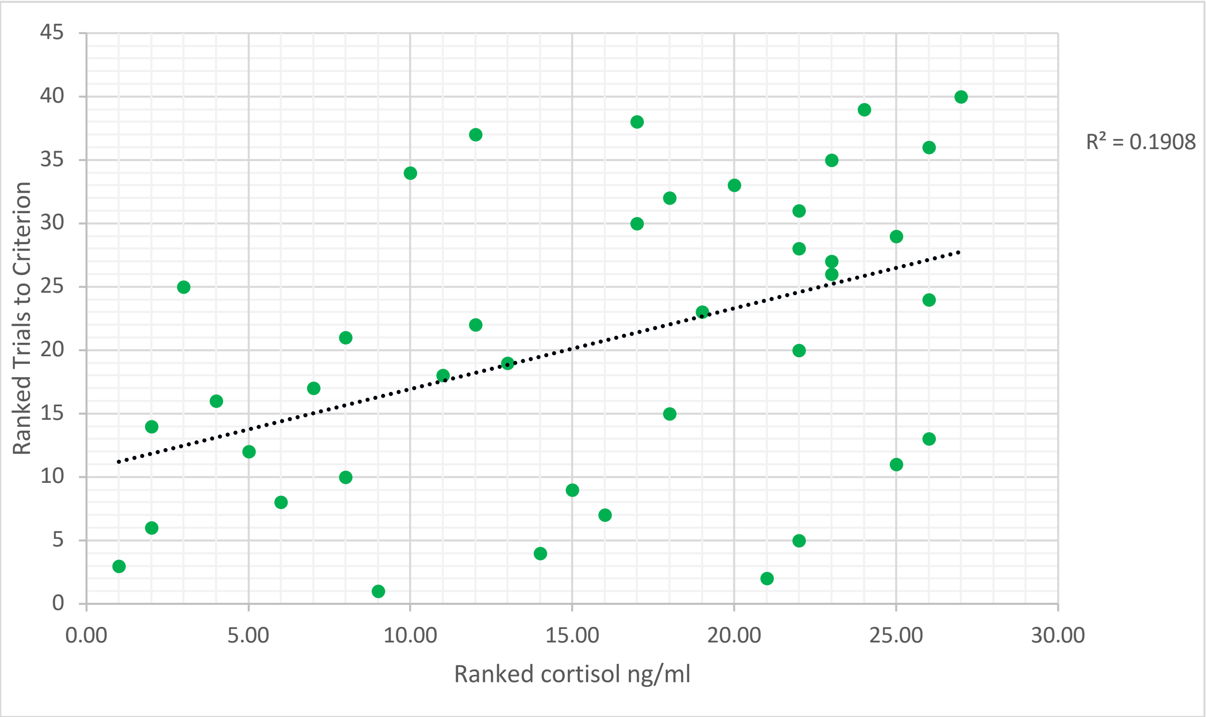

Supplementary Figure 2- Scatterplot of ranked trials to criterion v ranked cortisol concentrations during learning.
